# Metabolic markers of short and long-term exogenous DL-beta-hydroxybutyrate supplementation in episodic migraine patients: an exploratory analysis of a randomized-controlled-trial

**DOI:** 10.3389/fphar.2023.1172483

**Published:** 2023-05-04

**Authors:** Niveditha Putananickal, Elena C. Gross, Anna-Lena Orsini, Simone Schmidt, Patricia Hafner, Vanya Gocheva, Sara Nagy, Bettina C. Henzi, Daniela Rubino, Sabine Schädelin, Peter Sandor, Dirk Fischer

**Affiliations:** ^1^ Division of Neuropaediatrics, University of Basel Children’s Hospital, University of Basel, Basel, Switzerland; ^2^ Neurology, University of Basel Hospital, University of Basel, Basel, Switzerland; ^3^ Department of Clinical Research, Clinical Trial Unit, University of Basel Hospital, University of Basel, Basel, Switzerland; ^4^ RehaClinic, Bad Zurzach, Switzerland

**Keywords:** exogenous ketone bodies, ketone salt, DL-Mg-Ca-beta-hydroxybutyrate, migraine, long-term ketone supplementation

## Abstract

**Background:** Emerging findings propose that the pathophysiology of migraine may be associated with dysfunctional metabolic mechanisms. Recent findings suggest that migraine attacks are a response to the cerebral energy deficit, and ingestion of ketone bodies stabilizes the generation of a migraine attack. Based on these findings, ketone body supplementation is postulated as a prophylactic treatment approach to restore cerebral metabolism deficiency. Metabolic markers are unexplored after exogenous ketone body supplementation in episodic migraineurs. Therefore, the present single-arm uncontrolled explorative analysis evaluated blood ketone body and glucose concentration after short and long-term 6 g exogenous DL-Mg-Ca-beta-hydroxybutyrate (DL-βHB) supplementation.

**Methods:** The presented data are part of the MigraKet randomized-control cross-over clinical trial of 41 episodic migraineurs (Number NCT03132233). Patients were given a single dose of 6 g DL-βHB. Ketone body and glucose blood concentration were assessed before intake, 20, and 40 min after DL-βHB intake. Ketone body, glucose concentration and glycated hemoglobin values were evaluated after 12 weeks of 18 g DL-βHB ingestion (total dose), taken three times daily (6g/dose; 3x/day). Linear models explored the association between the ketone body and glucose levels.

**Results:** Ketone body concentration increased within-group to a mean of 0.46 (0.30) mmol/L after 40 min post- DL-βHB supplementation [estimate = 0.24 mmol/L, CI = (0.20.0.27), *p* < 0.01]. This within-group increase of ketone body concentration did not change after repeated daily intake of DL-βHB supplementation over 12 weeks [estimate = 0.00 mmol/L, CI = (−0.03.0.04), *p* = 0.794]. DL-βHB intake significantly reduced blood glucose concentration within-group from a mean baseline of 4.91 (0.42) mmol/L to 4.75 (0.47) mmol/L 40 min post-DL-βHB supplementation [estimate = −0.16 mmol/L, CI = (−0.15, 0.03), *p* < 0.01]. Repeated DL-βHB supplementation for 12 weeks showed no change within-group in acute ketone bodies concentration [estimate = 0.00 mmol/L, CI = (−0.03.0.04), *p* = 0.794] and in the HbA1c value [estimate = 0.02, CI = (−0.07.0.11), *p* = 0.69].

**Conclusion:** A single dose of 6 g DL-βHB significantly elevated blood ketone bodies and decreased blood glucose concentration within-group in episodic migraineurs. Long-term DL-βHB supplementation for 12 weeks showed no effect within-group on acute ketone body concentration and had not impact on HbA1c. The elevation of the ketone body concentration was moderate, indicating that nutritional ketosis was not reached. Therefore, a dose higher than 6 g of DL-βHB is required to reach the nutritional level of ketosis. ClinicalTrials.gov Identifier: NCT03132233.

## 1 Introduction

An increasing number of findings indicate that migraine pathophysiology may partially be associated with metabolic abnormalities causing cerebral energy deficits (Sandor et al., 2005a; Sandor et al., 2005b; Sparaco et al., 2006; Yorns and Hardison, 2013; Colombo et al., 2014). Prevalent migraine triggers, such as skipping meals, fasting, dehydration or sleep deprivation that can precipitate migraine attacks, are associated with deficient cerebral energy metabolism (Borkum, 2016; Gross E. C. et al., 2019). The molecular mechanism behind these triggers includes higher oxidative stress, increased cerebral excitability, hypometabolism, reduced glucose metabolism and transport, and mitochondrial dysfunction (Sparaco et al., 2006; Colombo et al., 2014; Borkum, 2016; Gross E. C. et al., 2019).

To amend the impaired metabolic state of the brain, the elevation of ketone bodies (KB) through a ketogenic diet (KD) or ketosis has been proposed and implemented, allowing the energy-deficient brain to utilize KB as an alternative energy substrate in the metabolic pathways (Maggioni et al., 2011; Di Lorenzo et al., 2015), thereby increasing glucose transport and cerebral metabolism, decreasing cerebral excitability and alleviating oxidative stress (Hartman et al., 2007; Ma et al., 2007; Stafstrom and Rho, 2012). Exogenous KB supplementation has been proposed in migraineurs to circumvent prolonged periods of fasting or stringent KD (Gross E. C. et al., 2019). This feasible method induces nutritional ketosis while maintaining the beneficial aspects of KD. We have implemented this non-restrictive approach in a previous randomized clinical trial that assessed the prophylactic effect of exogenous DL-Mg-Ca-beta-hydroxybutyrate (DL-βHB) supplementation in episodic migraineurs (Gross E. et al., 2019). No clinical attenuation of migraine frequency and intensity was reported in the trial (Putananickal et al., 2021).

Previous findings of exogenous KB administration in humans, specifically of ketone salts, suggest an acute blood ketone elevation reaching mild to medium nutritional ketosis (0.4–1 mmol/L), irrespective of blood glucose level (Chiolero et al., 1993; Gautschi et al., 2015). Further findings indicate that the most abundant KB, beta-hydroxybutyrate is also able to modulate glucose metabolism (Newman and Verdin, 2014). Furthermore, KB supplementation is also considered for its glucose-lowering effect (Stubbs et al., 2017; Falkenhain et al., 2022). The response of metabolic markers such as blood KB and glucose concentration after exogenous KB supplementation has not been assessed in migraine patients.

Therefore, this explorative analysis investigated the pharmacokinetics of the exogenous DL-βHB supplementation in episodic migraineurs. The primary objective was to assess the effect of 6 g exogenous DL-βHB supplementation on blood KB concentration. The effect of exogenous DL-βHB supplementation on blood glucose concentration was assessed as a secondary objective. In addition, the association between KB level and glucose level after DL-βHB supplementation was studied. Further, the effect of prolonged supplementation with DL-βHB was evaluated on acute KB and glucose concentration, as well as glycated hemoglobin (HbA1c) level.

## 2 Materials and methods

### 2.1 Trial design

The presented results utilize data from the randomized, double-blind, placebo-controlled, cross-over trial evaluating the efficacy of DL-βHB on migraine frequency and intensity in patients with episodic migraine. The study was registered at ClinicalTrials.gov (NCT03132233), study protocol was approved by the local Ethics Committee northwestern and central Switzerland (EKNZ 2015-304) and the National Swiss Drug Agency (2016DR2109). The study was conducted at the University Hospital Basel (USB) Switzerland. All patients provided written informed consent of study participation.

**FIGURE 1:**
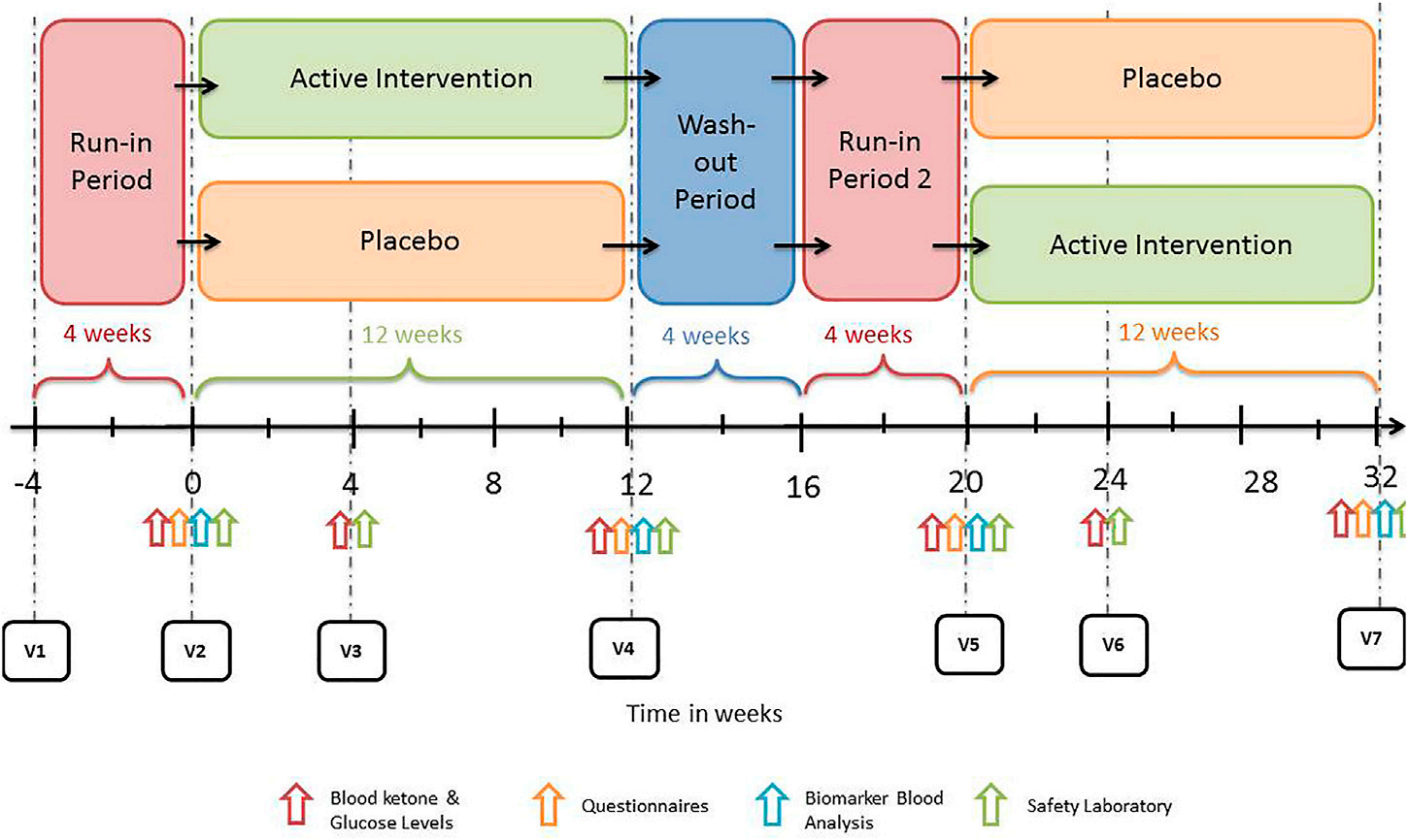
Flowchart of study design.

The main trial consisted of 4 weeks long run-in phase followed by a baseline visit (visit 1), then a first treatment period of 12 weeks (visit 4), followed by a washout period of 4 weeks and a second run-in phase of 4 weeks (visit 5) continued with the second treatment period of 12 weeks (visit 7). The trial overview is presented in Figure 1. An elaborate explanation of trial design is included in the published trial protocol (Gross E. et al., 2019).

### 2.2 Patients

Patients were part of the clinical trial conducted at the USB in Switzerland. Recruitment took place from May 2017 till September 2019. Patients were eligible for inclusion if aged between ≥18 and ≤65 years, had episodic migraine with or without aura assessed by the international classification of headache disorders version 3 Beta Classification (ICHD-3) criteria, onset of migraine occurred before age 50 years; experienced 5 to 14 migraine days per 4 weeks, refrain from initiating/changing the type, dosage/frequency of any prophylactic medications and dietary supplements (such as Q10, riboflavin etc.) against migraine during study and 4 months before study start, refrains to make any drastic changes to the diet for the duration of the study, including periods of fasting. The exclusion criteria were: no requirement of oral/injectable steroids, current intake of simple analgesics or non-steroidal anti-inflammatory drugs more than 14 days per 4 weeks; takes triptans over 10 days per 4 weeks for headaches or other body pain; intake of prescription opioids, previous diagnosis of medication overuse headache, which has reverted to episodic migraine within the last 6 months, meets the ICHD-3 Beta Classification criteria for chronic migraine. A detailed description of exclusion and inclusion criteria is provided in the published trial protocol (Gross E. et al., 2019).

### 2.3 Investigational product

In this clinical trial, the KB DL-βHB in powdered calcium-magnesium-salt form (Ca-Mg-DL-βHB) was used as the investigational medical product (IMP). The reason for this choice was that only pharmaceutical grade ketone salts were available in Europe at the time of trial implementation. One dose of Ca-βHB contained 2.51 g βHB and 0.49 g Ca^2+^. The daily dose of 9 g Ca-βHB contained 7.54 g of βHB and 1.47 g Ca^2+^ and was divided into three servings. Furthermore, one dose of Mg-βHB contained 2.2 g βHB and 0.26 g Mg^2+^. The daily dose was 9 g Mg-βHB contained 6.6 g βHB and 0.77 g Mg^2+^ and was divided into three servings. One dose per serving consisted of 3 g of Ca-βHB and 3 g of Mg-βHB. The IMPs were water-soluble powder. To maintain blinding, patients consumed the IMP diluted in water and combined with sugar-free syrup.

### 2.4 General visit procedures

Described here are procedures relevant to the metabolic marker assessment after DL-βHB supplementation. The published protocol describes the remaining procedures (Gross E. et al., 2019). After the screening visit, patients were randomized to either the placebo or the treatment arm (visits 2/3/4 or 5/6/7). Patients were asked to come in an overnight fasted state to each study visit. An unblinded study nurse measured KB and glucose blood levels during each visit after the safety blood test (baseline/t1). Patients then immediately consumed the IMP orally, diluted with water and sugar-free syrup. After 20 min, the blinded study nurse again assessed the KB and glucose blood levels (t2). This was followed by the last measurement 40 min (t3) after IMP ingestion. Water was freely permitted, and patients remained sedentary at the outpatient clinic throughout the study visit. Compliance was measured by collecting and counting the used and unused IMP sachets at each visit.

### 2.5 Measurement/blood collection and processing

This study’s pharmacokinetics evaluation was analyzed using the single-dose administration data. KB levels were measured using the finger-prick method by collecting capillary blood with a point-of-care blood ketone meter (precision xtra ™, Abbott) at each study visit (visit 2-7). This measurement allowed the detection of immediate changes in the blood KB levels. Glucose levels were assessed with a point-of-care blood glucose meter (precision xtra ™, Abbott), also allowing detection of immediate changes in the blood glucose. Additionally, HbA1c was assessed at each visit and analyzed at the laboratory of the USB. HbA1c represents the mean glucose levels over the last two to 3 months. Blood samples were immediately sent at room temperature to the inhouse laboratory of the University Hospital Basel for immediate analysis of HbA1c. HbA1c has the laboratory reference HbA1c 4.8%–5.9% (according to DCCT/NGSP).

### 2.6 Statistics

No statistical power calculation was conducted prior to the exploratory analysis. The sample size was based on the available data. KB concentration was measured during the first treatment period (visit 2- 4) and during the second treatment period (visit 5–7). The patient received the IMP, respectively, placebo during each treatment period. The KB concentration set consists of all subjects treated with the IMP (for visits 2/3/4 or 5/6/7). The sensitivity analysis set (SAS) consists of all subjects who have taken medication according to protocol. The glucose concentration set consists of all subjects who have valid glucose measurements. Again, only measurements under the IMP treatment were used (visit 2/3/4 or 5/6/7). Demographics and relevant baseline variables are summarized for patients with KB and glucose level assessments (Table 1).

**TABLE 1:** Characteristics of patients with blood ketone bodies and glucose assessments. Total migraine days refer to 4 weeks preceding baseline visit. Age in years. Migraine intensity (VAS 1-10), categorical data are presented as frequencies and percentages. For continuous variables, the mean and the standard deviation are presented. For skewed data the median and the lower and upper quartile are shown. No data was missing.

	βHB	Placebo
n	18	19
Age, y	32.3 (10.8)	36.4 (10.9)
gender = male (%)	0 (0.0)	3 (15.8)
Total migraine days	5.9 (2.3)	7.5 (3.0)
Migraine intensity, (VAS 1-10)	6.0 (2.0)	5.9 (1.9)
Migraine years	15.9 (10.3)	20.1 (10.7)

First, the IMP ingestion and its relation to KB were assessed. KB levels at 0, 20 and 40 min were analyzed in a linear model. Due to outliers, a robust model was used. Time points of measurement (0 vs. 2 min and 0 vs. 40 min) are included as fixed effect, subject as a random effect. It was assumed that ketone body concentration increases after medication. In addition, the same model was fitted as sensitivity analysis, including also variable visit (pooling visit 2/5, 3/6 and 4/7). In a second step, the interaction between visit and time was included, comparing the two models using a single ANOVA comparison. A significant effect of visit or the interaction would indicate pharmacokinetic changes with repeated IMP intake.

Next, the IMP and its relation to glucose concentration were analyzed. The glucose level at 0, 20 and 40 min were assessed as described above for blood KB levels. Since there were no outliers amongst the glucose level data, no robust estimator was needed. It was assumed that glucose level is reduced after initiation of IMP.

The association between blood glucose level and KB level at the same point was assessed in a linear model. Glucose level served as a dependent variable. Ketone body level and time point (20 or 40 min) were included as a fixed effect, study subject as a random effect. Furthermore, the association between glucose level and ketone body level at 0 min was assessed. However, in this analysis, ketone body level served as a dependent variable. It was assumed that there is an association between glucose and ketone body concentration. The HbA1c value at last visit (visit 5, respectively visit 7) was compared between IMP and placebo phase using a linear mixed effects model. Study subject was included as random effect.

For all models, model validation was performed by checking residuals and leverages. The residuals were checked by visual inspection using a plot of the residuals versus the fitted values and a Normal Q-Q plot. In the case of outliers, the analysis we performed a robust model. These analyses were performed on the ketone body concentration set and repeated on the SAS as sensitivity analysis. As described before, only measurements during IMP treatment were used.

All analyses are exploratory and descriptive. Results have thus to be interpreted as hypothesis-generating and not as confirmatory. *p*-values should be interpreted as a continuous measure of evidence against the corresponding null hypothesis (i.e., no association with predictor) and not as confirmatory (“significant” versus “non-significant”).

## 3 Result

**FIGURE 2:**
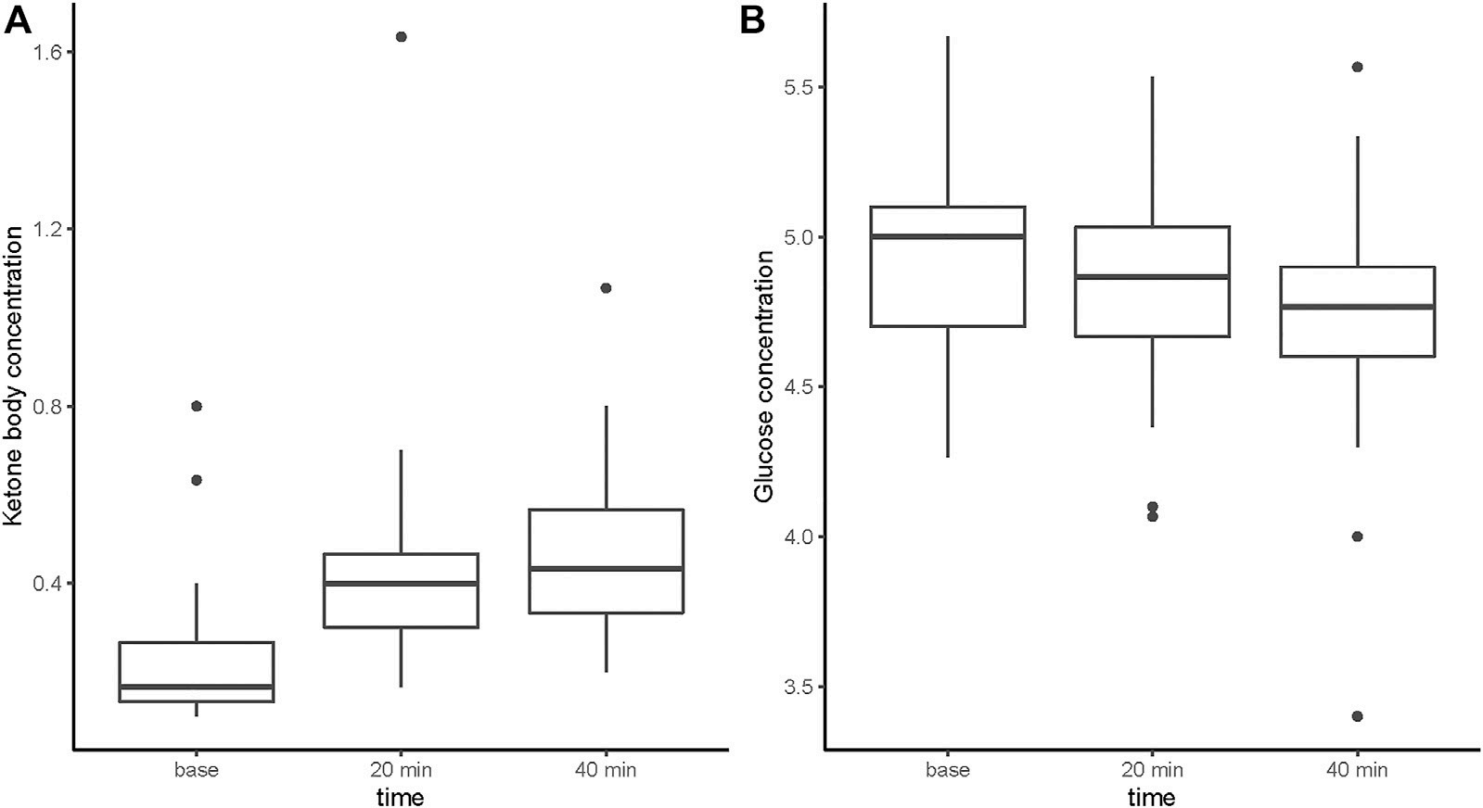
Blood ketone body and glucose concentration (mmol/L) following intake of βHB supplementation.

Demographics and relevant baseline variables of the patients with KB levels and glucose levels assessment are summarized in Table 1. Table 2 shows the mean blood KB and glucose level after the DL-βHB supplementation during the interventional phase of the trial, which are also presented in Figure 2.

**TABLE 2:** Summaries of glucose and ketone bodies blood levels in mmol/L after one dose of 6 g βHB. Represented are the mean values and standard deviation.

	Estimate	CI	*p*-value
KB level after 20 min (vs. at baseline)	0.19	[0.16.0.23]	<0.01
KB level after 40 min (vs. at baseline)	0.24	[0.20.0.27]	<0.01
Glucose level after 20 min (vs. at baseline)	−0.06	[-0.15.0.03]	0.17
Glucose level after 40 min (vs. at baseline)	−0.16	[-0.25,-0.07]	<0.01

The KB concentration increased 20 and 40 min after DL-βHB ingestion when compared to baseline concentration [20 min: estimate = 0.19 mmol/L, CI = (0.16.0.23), *p* < 0.01; 40 min: estimate = 0.24 mmol/L, CI = (0.20.0.27), *p* < 0.01] (see Table 3). No change was observed after 4 weeks of treatment compared to the baseline visit [estimate = 0.02 mmol/L, CI = (−0.02.0.06), *p* = 0.293] and the end of the intervention phase visit compared to baseline [estimate = 0.00 mmol/L, CI = (−0.03.0.04), *p* = 0.794]. Similar effects were observed in the SAS (see Supplementary Table S3).

**TABLE 3:** Treatment effect of one dose βHB on ketone body and blood glucose blood concentration in mmol/L.

	Baseline	20 min after IMP	40 min after IMP
Glucose	4.91 (0.42)	4.84 (0.42)	4.75 (0.47)
Ketone	0.22 (0.26)	0.43 (0.44)	0.46 (0.30)

The glucose concentration decreased 20 and 40 min after ingestion of DL-βHB when compared to baseline [after 20 min: estimate = −0.06 mmol/L, CI = (−0.15.0.03), *p* = 0.17; after 40 min: estimate = −0.16 mmol/L, CI = (−0.25, 0.07), *p* < 0.01] (see Table 3). Repeated intake for 4 weeks showed a significant increase [estimate = 0.10 mmol/L, CI = (0.01, 0.19), *p* = 0.03] compared to baseline. However, the repeated intake at the end of the intervention phase visit compared to baseline showed a decrease [estimate = −0.09 mmol/L, CI = (−0.18, −0.00), *p* = 0.0504]. These results were not consistent with the SAS (see Supplementary Table S4).

KB and glucose concentration assessed at the same time point did not show any association (see Supplementary Table S5). Further, no association between glucose and ketone level was detected at baseline [estimate = −0.00, CI = (−0.05.0.05), *p* = 0.90]. The same results were observed with the SAS (see Supplementary Table S6).

There was no change of HbA1c value during IMP intake compared to placebo at the end of each respective interventional phase [estimate = 0.02, CI = (−0.07.0.11), *p* = 0.69], again same results were observed with the SAS.

## 4 Discussion

This exploratory analysis of pharmacokinetics of exogenous DL-βHB supplementation in episodic migraine patients demonstrated four findings: (I) an increase of 0.24 mmol/L kB blood level after 40 min of the intake of 6 g of DL-βHB, (II) a clinically non relevant decrease of 0.16 mmol/L in blood glucose levels over 40 min after intake in comparison to baseline, (III) no change of the HbA1c value after 12 weeks of DL-βHB supplementation compared to placebo and (IV) no change in the short-term response of blood KB and glucose level after 12 weeks of DL-βHB supplementation.

The observed acute elevation in KB blood level after supplementation with DL-βHB was modest. This marginal elevation could be explained by the conservative dose selection of 6 g DL-βHB, which was defined to remain within the acceptable range of the mineral load to avoid severe gastrointestinal side effects. The modest KB elevation in our trial is in line with past reports of ketone salts supplementation in humans, which demonstrated similar low elevations in blood ketone concentration (O’Malley et al., 2017; Rodger et al., 2017). However, the study sample consisted of active cyclists compared to our sedentary patients.

Unfortunately, the increase in blood KB concentration achieved by DL-βHB supplementation was not comparable to the increase in blood KB concentration achieved by nutritional ketosis (≥0.5 mmol/L) (Harvey et al., 2018). This insufficient elevation could be attributed to the choice of the racemic mixture of DL-βHB, which was the only pharmaceutical-grade βHB available at the time of trial initiation. Our observations are in line with previous results of racemic DL-βHB supplementation in humans, reporting a similarly low elevation in blood ketone levels (Stubbs et al., 2017; Fischer et al., 2018). A possible explanation for the low increase is the higher elevation of blood L-βHB concentration compared with blood D-βHB concentration when DL-βHB is consumed (Stubbs et al., 2017). L-βHB is a non-physiological enantiomer of D-βHB. The latter is the natural and biologically active isomer. L-βHB is suggested to have a lower metabolic rate and higher elimination rate in urine when compared to D-βHB (Cahill Jr, 1970). Recent findings corroborated the notion of D-βHB possibly being an oxidative fuel, since exogenous DL-βHB intake did not elevate blood ketone levels as high compared to pure D-βHB intake (Cuenoud et al., 2020). Consequently, a higher dosage of the DL-βHB, pure D-βHB supplement or another ketone supplement like ketone esters should be administered to achieve nutritional ketosis exogenously (Stubbs et al., 2017; Falkenhain et al., 2022).

The observed a significant decrease of 0.16 mmol/L in blood glucose concentration at 40 min post- DL-βHB intake compared to before βHB intake was not as large as previous reports of KD and exogenous ketone supplementation treatment, which have demonstrated a glucose-lowering effect (Stubbs et al., 2017; Stubbs et al., 2018). Notably, a prior report of racemic DL-βHB supplementation also reduced the blood glucose level (Stubbs et al., 2017). Our observations could indicate that the uptake of exogenous KB could lead to the reduction of blood glucose concentration by reducing hepatic gluconeogenesis and increasing peripheral glucose level, even in presence of available carbohydrates (Mikkelsen et al., 2015; Stubbs et al., 2017). However, it should be considered that the observed decrease in glucose level could also be influenced by various factors such as physical activity, sleep, stress as well as the circadian rhythm.

Our findings showed no change in the short-term response of blood KB and glucose levels after 12 weeks of 6 g DL-βHB supplementation three times daily. The additional assessment of HbA1c value demonstrated no change as well over the 12 weeks of DL-βHB supplementation. Therefore, the chosen low dosage of DL-βHB was not able to induce a glucose-lowering effect that compound over time. Dose related changes have been observed in other ketone supplements, however with higher dosage (Soto-Mota et al., 2019). Consequently, our results implicate that long-term exogenous of 6 g ketone salts intake three times daily showed no marked differences in the glucose metabolism after 12 weeks.

Our findings have limitations. Firstly, the short observation period of merely 40 min after intake of βHB supplement. Consequently, only three time points (before intake, after 20 min, and after 40 min) were measured. It is uncertain if a longer observation period would have shown a higher increase in blood KB level, however a previous finding indicated that the intake of racemic DL-βHB resulted in a slow and moderate increase of blood KB level over 5.5 h in humans (Fischer et al., 2018). In our trial a longer period would not have been feasible because this would have resulted in prolonged fasting state of the patients. Future studies should therefore implement a longer observation period to assess the response to βHB supplementation. A further limitation is that the patients' dietary intake was not evaluated in our analysis. Patients were required to maintain their diet before the start of the study and during the study. Certain diets, such as low carbohydrate diet, may also alter KB concentration in the blood (Manninen, 2004). Therefore, we cannot exclude whether the diet of the patients had an impact on our results.

## 5 Conclusion

In episodic migraineurs, exogenous racemic DL-βHB supplementation resulted in the acute modest elevation of 0.24 mmol/L in blood KB levels corresponding to mild exogenous ketosis. However, the selected dose of racemic DL-βHB achieved only a marginal elevation of KB concentration when compared to KB concentration during nutritional ketosis. Furthermore, DL-βHB ingestion contributed to an acute significant blood glucose decrease of 0.16 mmol/L in migraineurs. In future trials higher racemic βHB doses or pure D-βHB supplementation should be considered to induce exogenous nutritional ketosis.

## Data Availability

The raw data supporting the conclusion of this article will be made available by the authors, without undue reservation.

## Abbreviations

HbA1c, glycated hemoglobin; ICHD-3, international classification of headache disorders version 3 Beta Classification; IMP, investigational medical products; KB, ketone bodies; KD, ketogenic diet; SAS, sensitivity analysis set; USB, University Hospital Basel; βHB, beta-hydroxybutyrate.
