# Supplementary material for: Metabolic markers of short and long-term exogenous DL-beta-hydroxybutyrate supplementation in episodic migraine patients: an exploratory analysis of a randomized-controlled-trial
Source: Front Pharmacol. 2023 May 4;14:1172483. doi: 10.3389/fphar.2023.1172483 (PMC10192563; doi:10.3389/fphar.2023.1172483)
Supplement: Supplementary file 1 [file Table1.DOCX]

***Supplementary Material***

Metabolic markers of short and long-term exogenous DL-beta-hydroxybutyrate supplementation in episodic migraine patients: An exploratory analysis of a randomized-controlled-trial

Niveditha Putananickal, MSc^1^, Elena C. Gross, PhD^1^, Anna-Lena Orsini, MD^2^, Simone Schmidt, MD^1^, Patricia Hafner, MD^1^, Vanya Gocheva, PhD^1^, Sara Nagy, MD^2^, Bettina C. Henzi, MD^1^, Daniela Rubino^1^, Sabine Schädelin, MSc^3^, Peter Sandor, Prof^4^, Dirk Fischer, Prof^1*^

^1^ Division of Neuropaediatrics, University of Basel Children's Hospital, University of Basel, Switzerland

^2^ Neurology, University of Basel Hospital, University of Basel, Switzerland

^3^ Department of Clinical research, Clinical Trial Unit, University of Basel Hospital, University of Basel, Switzerland

^4^ RehaClinic, Bad Zurzach, Switzerland

^*^Corresponding Author:

Prof. Dirk Fischer, Division of Neuropaediatrics, University of Basel Children's Hospital, Spitalstrasse 33, Postfach, Basel 4056, Switzerland.

Tel.: +41 61 704 22 72; Fax: +41 61 704 12 13; E-mail: dirk.fischer@ukbb.ch

Table 1: Sensitivity analysis set patient characteristics

|  | IMP | Placebo | Missing |
| --- | --- | --- | --- |
|  | 13 | 15 |  |
| age (mean (SD)) | 31.0 (9.7) | 37.7 (11.6) | 0.0 |
| gender = male (%) | 0 (0.0) | 1 (6.7) | 0.0 |
| Total migraine days (mean (SD)) | 6.2 (2.2) | 6.9 (2.9) | 0.0 |
| Migraine intensity (mean (SD)) | 5.9 (2.3) | 5.7 (2.0) | 0.0 |
| Migraine years (mean (SD)) | 12.5 (8.1) | 20.9 (11.6) | 0.0 |

Table 2: Glucose concentration set patient characteristics

|  | IMP | Placebo | Missing |
| --- | --- | --- | --- |
|  | 13 | 20 |  |
| age (mean (SD)) | 35.8 (10.6) | 37.8 (10.4) | 0.0 |
| gender = male (%) | 0 (0.0) | 3 (15.0) | 0.0 |
| Total migraine days (mean (SD)) | 6.4 (2.3) | 7.0 (2.9) | 0.0 |
| Migraine intensity (mean (SD)) | 6.1 (2.2) | 5.9 (1.8) | 0.0 |
| Migraine years (mean (SD)) | 17.6 (11.3) | 18.6 (10.8) | 0.0 |

Table 3: SAS of treatment effect on ketone body concentration including effect of visit

|  | Estimate | CI | p-value |
| --- | --- | --- | --- |
| 20 min vs baseline | 0.19 | [0.15,0.23] | <0.01 |
| 40 min vs baseline | 0.24 | [0.20,0.28] | <0.01 |
| visit 3/6 (vs 2/5) | 0.02 | [-0.03,0.06] | 0.466 |
| visit 4/7 (vs 2/5) | -0.01 | [-0.05,0.03] | 0.644 |

Table 4: SAS of treatment effect on glucose concentration including effect of visit

|  | Estimate | CI | p-value |
| --- | --- | --- | --- |
| 20 min vs baseline | -0.06 | [-0.16,0.04] | 0.21 |
| 40 min vs baseline | -0.18 | [-0.28,-0.08] | <0.01 |
| visit 3/6 (vs 2/5) | 0.08 | [-0.02,0.18] | 0.12 |
| visit 4/7 (vs 2/5) | -0.10 | [-0.20,0.00] | 0.06 |

Table 5: Association between ketone body and glucose concentration at the same point

|  | Estimate | CI | p-value |
| --- | --- | --- | --- |
| Ketone | -0.11 | [-0.23,0.01] | 0.08 |
| Ketone bodies at 20 min | -0.04 | [-0.14,0.05] | 0.3906 |
| Ketone bodies  at 40 min | -0.13 | [-0.23, -0.04] | <0.01 |

Table 6: SAS of Association between ketone body and glucose concentration at the same point

|  | Estimate | CI | p-value |
| --- | --- | --- | --- |
| Ketone | -0.11 | [-0.23,0.01] | 0.0813 |
| Ketone bodies at 20 min | -0.04 | [-0.14, 0.05] | 0.3906 |
| Ketone bodies  at 40 min | -0.13 | [-0.23, -0.04] | <0.01 |
